# “The kids get haggled over”: how institutional practices contribute to segregation in elementary schools

**DOI:** 10.3389/fsoc.2023.1250158

**Published:** 2023-10-31

**Authors:** Isabel Ramos Lobato, Alina Goldbach, Heike Hanhörster

**Affiliations:** ^1^ILS – Research Institute for Regional and Urban Development, Research Group “Urban Social Space”, Dortmund, Germany; ^2^Institute of Urban and Regional Planning, Faculty of Planning, Building, Environment, Technical University of Berlin, Berlin, Germany

**Keywords:** institutional discrimination, school segregation, school composition, institutional practices, school choice, educational inequality

## Abstract

School segregation is a key topic in urban, educational and inequality research. While previous studies have mainly focused on the effects of both parental school choice and residential segregation patterns on the composition of schools, we draw attention to institutional players steering access to elementary schools as one important dimension of institutional discrimination. Combining expert interviews with school principals and the local schools department with a quantitative survey among parents, we scrutinize the interplay between institutional structures and practices and parental school choice strategies. We identify three dimensions of institutional discrimination as being particularly relevant for school access, and thus for school segregation and inequality: a school’s guidelines and strategic objectives in dealing with segregation, the enrollment process, and a school’s profiling and information policies. These factors prove to be rather subtle, yet crucial facets of institutional discrimination, co-producing and perpetuating spatial inequalities.

## 1. Introduction

School segregation is a key topic in urban, educational and inequality research. Children from families with different socioeconomic and ethnic backgrounds are often more segregated in schools than in the neighborhood (Boterman et al., 2019). Such socio-economic and ethnic segregation in schools is a problem, since it is not only an expression of social inequalities, but also contributes to their reproduction (Sykes and Kuyper, 2013; Karakayalı, 2020; Mecheril, 2020). The questions of how school segregation arises and how the resulting educational inequalities can be addressed are therefore crucial.

Following its introduction in many European countries, parental choice has become a major driver of school segregation in many cities (Boterman et al., 2019; Wilson and Bridge, 2019). Based on the widespread tendency to associate a school’s composition with its performance, parental choice is increasingly informed by a school’s social and ethnic composition, thus often fueling school segregation. In various (European) countries, including Germany, the effects of both parental school choice and residential segregation patterns on the composition of schools have already been extensively studied (Bonal et al., 2019; Boterman et al., 2019; Candipan, 2019; Ramos Lobato and Groos, 2019; Dean, 2020).

However, parental choice and residential segregation patterns alone are not sufficient to explain school segregation and its underlying processes. Rather, previous studies show that institutional structures and practices, i.e., regulations and guidelines as well as their concrete institutional implementation, can also contribute to social and ethnic segregation both between (Ramos Lobato, 2017; Boterman and Ramos Lobato, 2022a) and within schools (Karakayalı, 2018). At the same time, institutional players, such as school principals, are also in part responsive to the preferences and practices of (middle-class) parents (Jennings, 2010). School segregation can thus also be understood as a consequence and expression of institutional discrimination (Karakayalı and Zur Nieden, 2019, pp. 888). We understand institutional discrimination as institutional structures and systemically embedded routines and practices in organizations contributing to the disadvantage and exclusion of social groups (Alvarez, 1979, p. 2). However, the ways in which institutional structures and practices influence school segregation still need to be explored in more detail. Our main objective is therefore to analyze how institutional structures and practices can influence school segregation (intentionally or unintentionally) and thus potentially reinforce inequality. We ask specifically how school departments and school principals steer access to elementary schools both directly through (internal) guidelines, routines, and practices, as well as indirectly by conveying values which may be perceived by parents as barriers and thus influence their school choice behavior. In our analysis, we identified three dimensions of institutional discrimination as being particularly relevant for school access, and thus school segregation and inequality: a school’s guidelines and strategic objectives in dealing with segregation, the enrollment process, and a school’s profiling and information policies.

To answer these questions, we focus on a neighborhood with a socioeconomically and racially diverse population in a large city in the German state (*Bundesland*) of North Rhine-Westphalia (NRW). Looking at five elementary schools located there, we find that student composition varies significantly from one school to the next, without mirroring the average composition of the surrounding neighborhood. Such a situation is quite typical of large European cities, where levels of school segregation are often significantly higher than residential segregation levels (Boterman et al., 2019). Empirically, our study combines both qualitative expert interviews (e.g., with school principals, the city’s schools department), and interviews with and a quantitative survey among parents. The resulting dataset allows us to scrutinize institutional structures and practices, parents’ school choice behavior, and how they influence each other.

## 2. Institutional discrimination shaping school segregation

Research on institutional discrimination shows that, in addition to parental choice, the institutional context, i.e., the strategies and practices of institutional players, also contributes to school segregation (Ramos Lobato, 2017; Voyer, 2019; Boterman and Ramos Lobato 2022a,b). Schools themselves play a vital yet ambivalent role at the operational level of organizations: they point to “a central paradox of the welfare state […]: they are conceptualized as mediators of inclusion into the relevant social systems, but at the same time they are exclusive themselves, in as far as they define their competence and refuse their services to certain individuals or even whole groups “(Radtke, 2003, p. 8). Cultural capital is strongly embedded in educational organizations and plays a crucial role in class reproduction and class distinction (Bourdieu, 1986). The “synergy between the cultural capital of middle-class parents and the cultural dispositions of the schools” (Bridge and Wilson, 2015, p. 497) thus constitutes a key mechanism in class advantage. It demonstrates that, instead of being the just reward of personal merit in education, educational achievement is a social construct.

Institutional guidelines such as those aimed at promoting academic achievements, can be, as outlined below, excluding mechanisms disadvantaging specific groups (Karakayalı, 2018; Karakayalı and Zur Nieden, 2019). Furthermore, the way these guidelines are appropriated, adapted and acted out at the organizational level, for example in a school’s enrollment processes, is relevant from an institutional discrimination perspective.

### 2.1. Institutions and organizations (co-)producing inequalities

Discrimination theories draw on racism research and the (intersectional) analysis of mechanisms contributing to social inequality (Scherr et al., 2017, VII). While the concept of racism is closely linked to individual prejudices or stereotypes, institutional discrimination reflects causal mechanisms and how the interests and taken-for-granted attitudes of the ‘white’ majority are inscribed in institutions (May, 2021, p. 1). To address and reveal these causal mechanisms, it is important to contextualize the analysis of organizations (and their respective structures, operating procedures and practices) in the political-institutional environment characterized by ideologies and power relations (Bhavnani, 2001, p. 9). Institutions have the “ability and power to do ascriptions” (Emmerich and Hormel, 2013, p. 14), classifying individuals into groups. Underlying these distinctions are certain expectations of normality, e.g., being proficient in the native language (Karakayalı and Zur Nieden, 2019, p. 890). These structures and practices are often based on historically evolved guiding principles and values.

Organizations such as companies, associations or schools have considerable inherent ‘potential’ for discrimination, with the application of formal regulations providing scope for unequal treatment (Hasse and Schmidt, 2012, p. 885). This discrepancy between “policies as written” (such as an institution’s guiding policies) and “policies as performed” (an institution’s practices and routines) is addressed by the concept of street-level bureaucracy (Lipsky, 1980). Norms and unclear regulations at the level of both institutions and organizations leave room for individual interpretations and thus also for discrimination. Regarding their task to translate unwritten or unclear guidelines into daily organizational routines and decision-making, front-line staff’s discretionary power can be understood as “forced discretion” (Hanhörster and Ramos Lobato, 2021, p. 14). Decision-makers in organizations follow rationalities and pragmatic self-interests in every single action, translating norms into guidelines for organizational structures and daily routines, and keeping workloads manageable: “The prerogative is the functioning of the organization, its effectiveness and its stability in time. Thus, non-inclusion follows the logics of avoiding expected difficulties or extra costs” (Radtke, 2003, p. 9). Research on institutional discrimination thus deals with uncovering these written or unwritten codes, guidelines and/or group distinctions in everyday organizational life.

Importantly, many of these inequality-producing structures and routines are not necessarily considered unjust or illegal. Organizations may discriminate against some of their members, respectively clients, “without an obvious malevolent intention, decision or declaration” (Radtke, 2003, p. 9). Feagin and Feagin (1986) introduced the distinction between direct and indirect institutional discrimination, defining direct discrimination as “organizationally-prescribed or community-prescribed actions which have an intentionally differential and negative impact on members of subordinate groups” (p. 30). This includes disadvantaging effects for specific social groups caused by practices and routines in the organizational culture which are embedded in legal and/or administrative regulations (May, 2021, p. 3). In many cases, characteristics such as migration background, religion or gender are used as a proxy to predict a client’s ‘fit’ in the established system in order to avoid complex organizational restructuring processes. Indirect institutional discrimination on the other hand addresses more covert forms of ‘*de facto*’ discrimination (Marvasti and McKinney, 2007, p. 68), for example apparently neutral policies or longstanding entrenched practices which seem ‘normal’ and which are not explicitly meant to harm specific individuals or groups (Dovidio et al., 2010, p. 10). However, they reproduce whiteness as a norm (Ahmed, 2012). Moreover, as a result of rules being applied to all customers or clients (for example language skills), “different groups may have fundamentally unequal chances of complying with them” (May, 2021, p. 3; Gomolla and Radtke, 2009, p. 281). Exploring the presence of institutional discrimination thus involves looking at the often very subtle interconnections of administrative processes and inequalities (Murji, 2017, p. 82; Lewicki, 2022).

Institutional discrimination as well as discriminatory climates in schools have been analyzed in various areas in recent years, ranging from the structure of teaching, *via* the assignment of grades, to recommendations for the transition to a secondary school (van Zanten, 2007; Jennings, 2010; Karakayalı and Zur Nieden, 2019; Voyer, 2019; Baysu et al., 2022; Dursun et al., 2023). In the following, we focus on the institutional mechanisms contributing to segregation in elementary schools.

### 2.2. The organization at work: school guidelines, admission policies, and profiling

By introducing market mechanisms into education, parents have become clients and schools providers of the product ‘education’: “In the case of the school, the general maxim of action and decision-making is to keep the classroom going” (Radtke, 2003, p. 13). In their everyday activities, stakeholders in schools have to navigate between partly contradicting explicit and implicit requirements and expectations. In elementary schools, this concerns conflicting expectations about offering equal opportunities to all children and the demand to increasingly act in conformity with the market. With regard to established structures and the efficient design of processes, clients are categorized as ‘problematic’ when they cannot be “treated within the ‘normal’ procedures” (Radtke, 2003, p. 13). A key factor of discrimination in the school context is native language competence and thus a student’s migration background. The attempts of schools to keep down the number of certain children seen as ‘problematic’ clearly show that discrimination is not only caused by individual stereotypes, but also originates in institutionally embedded processes of ‘othering’ (Spivak, 1985; Karakayalı and Zur Nieden, 2019, p. 900; May, 2021).

The scope for schools to influence their composition by attracting ‘desirable’ families, despite or precisely because of the increasing influence of parents, becomes clear at various levels: first, with regard to guidelines and how schools implement educational norms; second, with regard to the enrollment process; and third, in the context of targeted profiling and information policies.

(1) Overarching programs, policies, and (a lack of) guidelines can influence – explicitly or implicitly – school segregation. For example, a study from Malmö, Sweden (Voyer, 2019), illustrates the segregation-promoting impact of certain local schools department policies and strategies, including the relocation of specific schools to neighborhoods with lower levels of immigration or the closure of schools and their reestablishment with offerings and programs assumed to primarily attract white students. Similar measures are described by Green et al. (2022) in several US cities where school district policy decisions, such as the establishment of special programs and uneven investment, disinvestment, and reinvestment decisions, reinforce school segregation. One exception is the ambitious ‘anti-segregation shock plan’ in Barcelona, Spain, which aims to reduce school segregation by distributing socially disadvantaged children evenly among all public and state-subsidized private schools (Bonal and González Motos, 2023).

(2) Studies emerging from critical discrimination and racism research show that institutional discrimination or racism can further contribute to the uneven distribution of racialized students (Gomolla and Radtke, 2009; Fereidooni, 2011; Karakayalı, 2020; Steinbach et al., 2020). The introduction of school choice is often accompanied by greater autonomy for schools in admitting students (Jennings, 2010), enabling school principals and administrations to influence school composition in terms of a ‘good mix’. Studies show, for instance, a preference for non-migrant students and those from families with high socioeconomic capital, with whom many school principals hope to achieve better rankings and thus to improve their reputation. Moreover, the effort to achieve a ‘good’ mix must also be seen in the context of teachers’ efficacy. A study from Flanders (Van Eycken et al., 2022) shows that teachers’ emotional exhaustion in low-SES schools is higher, resulting in stronger intentions to change schools. At the same time, students with little knowledge of the local language or those with behavioral problems are considered ‘deficient’ (Gillborn and Youdell, 2000; West and Hind, 2003; Dursun et al., 2023). Looking specifically at the German context, Kemper and Supik (2020) show how the use of vague and partly subjective categories, such as ‘non-German language of origin’, ‘migrant experience’ or ‘immigrant history’, in the admissions process is reflected in official school statistics. Such categorization further contributes to an ‘othering’ of groups perceived as ‘foreign’ and ‘undesirable’ in access policies.

(3) Selective practices are not limited to the admissions process itself. In particular, those schools unable to selectively enroll students try to increase their attractiveness by offering special educational and support services tailored to specific social target groups (mainly middle-class households) (Breidenstein et al., 2020). Subjected to greater competitive pressure and concerned about potential budget cuts, school principals are becoming increasingly interested in enhancing the position and reputation of their schools (Jennings, 2010; Voyer, 2019). Using targeted changes in profiling, they seek to attract certain social groups and thus influence their school’s composition with a view to achieving the ‘right’ mix (van Zanten, 2007). In this context, Fuller-Hamilton (2019) also refers to so-called ‘gifted programs’: “Racialized policies, such as those that outline accessing gifted and honors classes, are tools used to continue to stratify racial groups and promote the status quo of ‘what’s white is right’” (p. 762).

Other studies also refer to targeted marketing by schools, e.g., through a school’s website, open days or information events, as well as targeted cooperation with strategically selected educational institutions with a similarly ‘privileged’ composition, such as kindergartens (van Zanten, 2013; Ramos Lobato, 2017). Besides those forms of marketing targeting specific groups, some schools also seem to resort to measures aimed at ‘deterring’ or ‘re-counseling’ students and racialized families perceived as ‘problematic’ (Ball et al., 1996; Gillborn and Youdell, 2000; West and Hind, 2003; Noreisch, 2007; Jennings, 2010; Voyer, 2019; Nast, 2020).

Looking specifically at Germany, institutional influence on school segregation has so far been researched less extensively and in less detail. Existing studies often relate to Berlin, a *Bundesland* where catchment areas regulate elementary school admission, and/or tend to focus on segregation within schools, i.e., between classes (Karakayalı, 2018). Consequently, there is limited knowledge on the influence of educational players on school segregation in other German states, particularly in those with free elementary school choice such as NRW. Against the background of increased parental choice, we thus ask how institutional practices can shape accessibility to elementary schools.

## 3. Case study and methodological approach

### 3.1. Setting the context: the German education system in a nutshell

Germany has a devolved education system, with each of its 16 federal states in charge of setting their own school regulations. The overall system is known for its comparatively high degree of social selectivity and inequality – especially due to the early (at the end of Grade 4 in most states) assignment of students to different secondary school forms dependent on their academic performance. In all federal states, elementary schools are the only schools where all children of a single age group are taught together. They are thus considered more egalitarian educational institutions than the (more) segregated secondary schools (Maaz et al., 2022). Nevertheless, parents increasingly consider the choice of the ‘right’ elementary school as a crucial first step in their child’s educational career (Ramos Lobato, 2019; Ramos Lobato and Groos, 2019; Breidenstein et al., 2020). Indeed, the transition from elementary to secondary school can have far-reaching effects, as a subsequent move from a lower-level to a higher-level secondary school form remains the exception (Bellenberg and Forell, 2012).

Parents can choose between differently funded elementary schools. Public schools are 100% publicly funded and do not charge tuition fees, whereas private schools are on average 75% publicly funded (Klemm et al., 2018, p. 29). The proportion of students attending private schools is increasing throughout Germany, though their overall share of 3.6% in elementary schools is less than in many other European countries (Grossarth-Maticek et al., 2020). In many cases, private elementary schools are based on a specific religious or pedagogical principle, while state denominational elementary schools (Catholic and Protestant) exist solely in the states of NRW and Lower Saxony. They are fully publicly funded and tuition-fee-free but at the same time allowed to prioritize children with the ‘right’ denomination (Landtag Nordrhein-Westfalen, 2016). In NRW, state denominational elementary schools account for around 30% of all public elementary schools (Ministerium für Schule und Bildung des Landes Nordrhein-Westfalen, 2022).

To enable efficient planning and ensure short distances between home and school, for decades access to public elementary schools was organized through catchment areas in Germany. However, unlike most other states, NRW abolished these in 2008 and introduced elementary school choice. Theoretically, the policy reform enables parents to register their children at any elementary school. Nevertheless, there is still a legal entitlement to a place in the nearest school “in accordance with a school’s predefined capacities” (Schulgesetz NRW §46 Absatz 3). Apart from spatial proximity, further criteria regulating access to elementary schools exist, but seem to be rather fuzzy. The final decision on the admission of pupils is up to the principals, who are also allowed to reject pupils if the school has insufficient capacity (Schulgesetz NRW §46.1). Since information on ‘school quality’ such as rankings or school performance is not published in Germany, school websites and open days are the main ‘official’ information sources for parents’ choices.

Compared internationally, levels of school autonomy and accountability as well as the frequency of mandatory standardized tests at school are less pronounced in Germany and significantly below OECD levels (OECD, 2016). School achievement data is usually not posted publicly, school rankings do not exist, and schools receive uniform funding per pupil. It is precisely this circumstance that makes the German context so intriguing. Considering the low level of school accountability, it is relevant to analyze institutional practices of controlling access to schools and, subsequently, school composition. NRW, a *Bundesland* where school catchment areas have been abolished, serves as an interesting case to explore to what extent free school choice can fuel competition between schools even in a system where performance-based funding cuts are the exception and accountability pressure is low.

### 3.2. The case study area Treefield

As a case study, we chose ‘Treefield’,^1^ a neighborhood in a large city in NRW. Treefield’s population can be considered as socially mixed: the proportions of welfare recipients (8.4%) and of people ‘with a migration background’^2^ (52%) are comparable with urban averages in Germany. Treefield has five public elementary schools: three non-denominational ones (one of which is a branch of an elementary school in another neighborhood) and two public denominational ones (one Catholic and one Protestant).

Conducted beforehand, the quantitative analysis of official structural data of our case study illustrates that student composition in the elementary schools deviates significantly from the neighborhood’s residential composition. However, this is by no means the exception: it reflects the relationship between school and residential segregation in many national and international urban areas (Boterman et al., 2019) as well as in our case study city. Besides the difference between neighborhood and school composition, we also find large composition differences between the schools. Used as an indicator of a child coming from a low-income household, the share of students exempted from paying for learning material^3^ ranged from 3.4 to 50.9% (compared to 16.2% for all Treefield schools). Moreover, the analysis of recent enrollment figures (i.e., information on parents’ first choice) reveals the different levels of popularity of the five schools (see Table 1). In recent years, especially those schools where children with a migration background or exempted from paying for learning material were underrepresented tended to be over-enrolled. Chestnut School appears to be an outlier in this context, though it too suffered from overcrowding in the past. This was overcome through its advertising efforts, as discussed below.

**Table 1:** Overview of the schools’ popularity and composition in the case study area compared to the neighborhood’s composition.

School	Proportion of children exempted from paying for learning material	Proportion of children with a migration background	Popularity measured by enrollments in relation to enrollment capacity
Treefield (total)	16.20%	52.19%	
Poplar school	50.94%	70.37%	Under-enrollment / low popularity
Aspen school	28.19%	63.64%	Previous under-enrollment / now balanced
Chestnut school (Catholic)	10.39%	43.65%	Previous over-enrollment / now balanced
Maple school	12.25%	56.63%	Over-enrollment / high popularity
Birch school (Protestant)	3.41%	36.27%	Over-enrollment / high popularity

Own calculation.

### 3.3. Data collection and analysis

The results presented here belong to a broader empirical research project analyzing the spatial, institutional, and individual dimensions of school segregation. In this article, we focus on the institutional dimension. To scrutinize how both the city’s schools department and school principals steer access to elementary schools directly through (internal) guidelines, routines, and practices, we draw on (11) qualitative interviews with officials from the schools department, all Treefield elementary school principals as well as heads of selected kindergartens and a migrant parental organization. These interviews were framed by several documented meetings with representatives from the schools and schools department as well as an analysis of the schools’ media presence and publicly accessible internal documents (e.g., enrollment documents) provided by the schools department.

To be able to capture and analyze the indirect ways used to steer access to schools, we also conducted a quantitative online survey among all parents of first and second graders in the neighborhood (331) and qualitative interviews with 55 parents,^4^ some of them were recruited through the quantitative survey. Both survey responses and parent interviews serve to contextualize the perspective of institutional players by making clear how strongly parents are guided in their school choice by institutional framework conditions and how closely parental choice and the practices of institutional players interact. Parent interviews also helped assess stakeholders’ statements in relation to their actions, as “what people say is often a poor predictor of what they do” (Jerolmack and Khan, 2014, p. 178). The attitudes or guidelines expressed by stakeholders are thus not necessarily reflected in their concrete practices. In particular, the impact of school practices was captured through the perspective of parents.

We aimed at capturing the perspectives of parents from different social situations and backgrounds. A key challenge, however, was that the fieldwork took place during the pandemic, when much of the teaching was online. We recruited parents in several ways, approaching them through school stakeholders (parent representatives, class teachers, principals), attending parents’ evenings and standing outside school gates on several days at drop-off and pick-up times to talk to parents and hand out flyers. Despite these efforts conducted over a period of 2 months, parents with low social status and migrant backgrounds are unfortunately still under-represented in the study. However, we are aware of this bias and took it into account when analysing the data.

All expert interviews focused on organizational structures, guidelines and practices influencing access to local elementary schools (e.g., enrollment processes and information policies). The parents were asked about their school choice criteria, their information sources, the enrollment process, and their conceptions of schooling, including the significance of active choice-making. All qualitative interviews were semi-structured, lasted between one and 3 hours and were conducted *via* videoconference between February and May 2020. They were recorded, transcribed, coded and analyzed following the principles of content analysis according to Mayring (2015).

To promote reflexivity and dialogue within the research team, to ensure coding consistency and to arrive at similar findings (O’Connor and Joffe, 2020), the team of coders met weekly during the coding phase. In a first step the coding list was developed deductively from the theory, while in a second step it was inductively supplemented. In addition, the team coded several interviews in parallel. To reach a common understanding, sections in which codings differed were discussed and the codes either revised or subsequently specified in more detail. The joint coding and analysis within the research team as well as the discussion of key project findings in a workshop attended by all Treefield school principals and key stakeholders from the local and regional schools administration and the local statistics office served as quality assurance in terms of a multi-perspective analysis of the interview data.

## 4. Institutional discrimination or equal access to schools? Institutional guidelines and practices and their impact on school segregation

In our analysis, we scrutinize how institutional school structures and practices can determine access to elementary schools – both directly through (internal) guidelines, routines, and practices, as well as indirectly by conveying values and boundaries that are perceived to influence school choice behavior. We are thus interested in whether and how institutional players intentionally or unintentionally reinforce school segregation and inequality. In addition, we pay attention to the interplay of institutional and parental practices by asking both to what extent institutional structures and practices influence parental school choice and, conversely, how (anticipated) parental preferences influence schools’ strategies and profile setting. In doing so, we were able to identify three key dimensions of institutional influence, according to which the following chapter is structured: a school’s guidelines and strategic objectives in dealing with segregation (4.1), the enrollment process (4.2), and a school’s profiling and information policies (4.3).

Access to elementary schools, and thus their composition, is influenced in two ways: first, by overarching guiding principles and regulations (or the lack thereof) – the so called “policies as written”; and second, by their concrete implementation, particularly in the context of the admissions procedure – the so called “policies as performed” (Lipsky, 1980). This means that overarching guidelines and strategic goals in dealing with segregation not only reflect theoretical ideas about the social composition of schools, but also convey social values and thus have a tangible effect, for example, on whether people feel welcome or not, thereby promoting or preventing institutional discrimination.

### 4.1. (Lacking) guidelines on school composition and schools’ understanding of a ‘good’ mix

Interestingly, our analysis of the interviews with representatives of the city’s schools department and the Treefield school principals and of municipal documents (e.g., the school development plan) reveals that there are no explicitly defined common guidelines or shared goals on the social composition of schools, whether at city or neighborhood level. These initial findings are corroborated by Boterman and Ramos Lobato’s (2022b) comparative study of school choice policy reforms in the Netherlands and Germany, demonstrating that even school reforms specifically designed to re-regulate school admissions procedures address neither the (desired) composition of schools nor segregation in schools *per se*. The lack of overarching guidelines or guiding principles on the social composition of schools means that school principals (have to) fill this gap with individual decisions and concepts. However, previous research has shown that lacking or fuzzy guidelines are particularly detrimental to those who are already disadvantaged (Allard and Small, 2013). Therefore, the question arises whether and to what extent the institutional practices of school principals are shaped by their implicit and/or individual concepts and perceptions of the ‘right’ school composition.

What we found is that such implicit views on school composition were indeed evident in our interviews, with school principals and schools department officials indirectly describing what they understand to be a ‘good’ mix, as for instance stated by the principal of the previously less popular Aspen school:

SP: “*What we […] have noticed […] [is that] our parent composition has developed very, very, very […] for the positive […]”.*

I: “*What do you mean by […] developed for the better […]?”*

SP: “*We now have many lawyers, doctors, people with very high academic degrees among our parents […]. From the professional image, from the appearance, you can see that a change has really taken place.”* (SP Aspen School)

As shown in previous studies (Noreisch, 2007; Jennings, 2010; Van Eycken et al., 2022), attracting parents with high educational attainment levels is usually described as positive and/or beneficial for a school and for teacher efficacy and turnover rates. Likewise, the school principals considered a certain ‘appearance’ of parents as an indicator of a ‘good’ mix. In addition, the fact that in recent years parents in ‘good employment’ had opted for her school was described as a positive development by the principal of the less popular Poplar School – and thus as a sign that the school did something right. The positive development of recent years was contrasted with earlier student populations, as in the example of the previously less popular Aspen School:

*“We used to have […] a lot of children with a migration background. […], where […] there were problems, I would say, with the language. Where problems had a lot to do with arriving in our culture group, as we know it. […] So, though we now still have children with a migration background, many of them are Asian […]. And we also have other children with a migration background, but where you don’t notice it at all. […] In the past, […] many mothers were still wearing headscarves and relatively long robes.”* (SP Aspen School)

In this quote, the principal relates the less positively perceived previous school composition to specific racialized groups or their appearance. Similar to previous studies, the quote illustrates a rather deficit-oriented perspective on children’s home language (Dursun et al., 2023), the intersectionality of different categories, such as race, ethnicity, religion, and social status (Scherr et al., 2017), as well as the different hierarchies of migrants – with parents with an Asian background and non-Muslim faith being seen more positively than Muslim parents. Moreover, it shows that ‘cultural’ assimilation of those categorized as migrants is interpreted as a ‘positive’ development (“*children with a migration background, but where you do not notice it at all”*). Other principals assess their schools’ social composition in a similar way, enabling conclusions to be drawn on more implicit guiding principles about the ‘right’ school composition. The principal of the Protestant Birch School, a school with a history of overcrowding, a socio-economically privileged and generally white/German composition (see Table 1), differentiates between children with a ‘migration background’ whose parents consider it important to ‘integrate’ (who, according to the principal, specifically choose the popular Birch School), and those whose parents prefer to be ‘among foreigners’ and, subsequently, would not choose Birch School. This narrative reveals a certain integration paradigm in the sense of expecting people with a migration background to assimilate into the ‘majority society’, an aspect also visible in a similar form at the other schools. This contrasts with an understanding of integration as cultural, social and symbolic belonging and recognition of diversity in all spheres of society, an understanding which has become more firmly anchored in Germany in recent years, both academically and politically (Foroutan and Kalter, 2021, p. 72, similar also in Foroutan, 2013; Die Beauftragte der Bundesregierung für Migration, Flüchtlinge und Integration, 2021).

However, it is important to mention that the above excerpts are not necessarily to be interpreted as evidence of principals’ individual prejudices or to problematize their choices of words, but rather to illustrate how racialized students are discussed in the institutional discourses surrounding school composition. They also show that society-wide embedded ‘racist knowledge’ (Terkessidis, 1998) or anti-Muslim logics (Karakayalı, 2020, p. 8), also to be found in other societal realms, play a certain role in education. Therefore, such logics are not present solely in institutional discourses. As has already become clear in various international studies (Byrne, 2006; Vowden, 2012; Ramos Lobato, 2019), we find similar narratives and ideas of a ‘good’ mix at schools as well as a similar differentiation between ‘good’ and ‘bad’ migrants among parents. Moreover, institutional and parental discourses most likely influence each other.

At the same time, it is worth noting how the principals of those schools where children with a migration background or from low-income families are clearly underrepresented in comparison to the neighborhood composition interpret their ‘mix’. The head of the popular Chestnut School, the Catholic school, for example, describes the composition in the following manner:

*“We have a healthy, a good mix here. Yes. It is not, I say, an elitist, snobby school, […] but also not a deprived school…that’s why I think […] that the parents […] have the feeling that we as a school cope well with this, […]diversity.”* (SP Chestnut School)

Here again, and thus not solely in the schools with a more privileged composition, diversity is not described as normality or enrichment, but rather in a deficit-oriented way: as something one has to ‘cope with’. The rather deficit-oriented perspective on diversity does not go unnoticed by some parents, as the following quote from Sinem^5^ (Turkish-German with a PhD) makes clear:

*And there was an event in the afternoon care that I don’t think was so good […] There was a child from the third grade who spoke Turkish and my daughter answered in Turkish […] And then the teacher said that Turkish must not be spoken because she wouldn’t know if they were saying something bad […] I thought that was very wrong, because it means my daughter is taught that speaking Turkish means something bad. If the child had spoken English, people would have said, ‘Oh great, the child already speaks English like her own native language. She doesn’t even have to learn it anymore.’”* (Sinem)

Even though this mother had already made her choice, her experience and assessment may well have an impact on the school choice of other parents. According to our survey, other parents are the most frequently named source of information for guidance in school choice (87%). This means that not only hearsay, but also very tangible personal experiences of parents are passed on and considered for school choice. In addition, some of the parents in our sample were rethinking their choices after their experiences with their first child when choosing a school for their second child. Thus, the rather deficit-oriented perspective on diversity can also have a self-reinforcing effect. Some parents – both with and without a migration background – emphasized the potential of diversity at schools, and therefore deliberately avoided those schools not recognizing this potential.

Interestingly, when school principals and schools department officials described a ‘good’ mix, little connection was made to the neighborhood’s social composition. A ‘good’ mix would seem to be one in which racialized students or students from poorer households are present, but only in significantly low proportions – i.e., even if this means that the proportion is way below the corresponding proportions in the city as a whole or in the neighborhood. Consequently, the social mix also tends to be addressed at school level rather than across schools. In the interviews, segregation was usually interpreted as a problem of the less popular schools rather than a neighborhood-wide challenge also affecting the popular schools where children with a migration background or from low-income families were underrepresented. One schools department interviewee put it this way:

*“[…] the system that we are running, at least from our point of view, is fair and easily comprehensible […] Certainly, we as the schools department, together with the school supervisory board, have to keep on checking whether we can strengthen certain schools, such as [names school with low enrollment numbers] and how we can change the image of any such school.”* (schools department representative)

The quote illustrates that, in line with a more market- and competition-oriented understanding of education (Makris, 2018), dealing with segregation is understood as a clear responsibility of the less popular schools. Rather than providing overarching guidelines on school composition, the responsibility for school composition (and segregation) is passed on to the school. Moreover, it seems to be solely left up to the more disadvantaged schools to change segregation patterns by increasing their popularity and boosting their reputation. Yet the latter is directly associated with a school’s social composition. Consequently, the more popular schools do not see any need to take action (or at least this was not addressed in the interviews) to promote access for children with a migration background or from a low-income family.

The lack of overarching guidelines not only affects the composition of schools and thus the prevention/mitigation of segregation, but also the targeted support of disadvantaged schools through a needs-based allocation of resources. Based on a school social index (calculated using data at the level of cities and districts), additional funds in the form of school social workers are distributed to particularly disadvantaged schools in NRW. However, this index is not sufficient for targeted resource allocation as it does not reveal the very different situation of individual schools within neighborhoods and cities. This is confirmed by the principal of the unpopular Poplar School who complained about the pressure on her school and who drew a connection to resource distribution: The allocated positions for social workers had so far not met her school’s specific needs^6^. On the part of the schools department, an allocation of resources in the sense of a targeted strengthening of schools with a high proportion of children from low-income families was discussed, but was not seen to be politically enforceable:

*“Years ago, we tried […] to distribute municipal services differently. We failed because of the politics […]. They said, ‘No one will be deprived of anything, everyone who has something [laughs] will keep it’. So, it can only be about new resources. And there was no money for that. […] Once things have been distributed, it is extremely difficult to change them afterwards.”* (schools department representative)

Overall, the interviewees only sporadically addressed inter-school relationships. The principal of the comparatively unpopular Aspen School was the only one to critically note that a ‘two-tier school system’ had emerged between ‘hotspot schools’ and schools with ‘extreme over-enrollment’. She called on the schools department to make greater efforts to achieve greater ‘mixing’ – without, however, explicitly calling for a cross-school or neighborhood-oriented strategy.

### 4.2. The enrollment process: “*Think of it as an Arab bazaar”*

Since the majority of Treefield’s elementary schools are running at full capacity, some schools are forced to turn down children. However, school enrollment data shows that, while between 2018–2020 approximately 10% of applications were rejected across all schools in the neighborhood, places were always available in the less popular Poplar School. Thus, Treefield’s growing population is not solely responsible for over-enrollment in certain schools. Parents’ school preferences also play a role. Nevertheless, the more important questions are: How do schools deal with over-enrollment and how does this affect school segregation? And, subsequently, to what extent do these practices promote inequality and are associated with institutional discrimination?

The basis for deciding whether to accept students at the more popular schools is governed by various criteria set forth in NRW school legislation in the regulation ‘*Ausbildungsordnung Grundschule’* (Ministerium des Inneren des Landes Nordrhein-Westfalen, 2005). This regulation states: “Every child has the right to be admitted to the elementary school of the desired type in his or her municipality which is closest to his or her home, within the limits of the admission capacity determined by the schools department.” Thus, in the case of over-enrollment, spatial proximity and school type (in Treefield’s case, the denominational or non-denominational orientation of the schools) are the decisive criteria. The latter clearly leads to non-Christian children having fewer chances to attend popular denominational schools – which is, despite being enshrined in the North Rhine-Westphalian constitution, a clear form of direct institutional discrimination. This has a direct impact on the schools’ composition: While the proportion of Protestant/Catholic children is higher at the respective denominational schools than at the other schools, the proportion of Muslim children is significantly lower. According to our quantitative analysis, the negative relationship between having a ‘migration background’ and the probability of being accepted at the chosen elementary school is weak but significant. Importantly, our parent interviews reveal that this relationship is not solely based on schools’ admission regulations, but also on parents’ choices and how they influence each other. According to several interviewees, out of fear of being rejection some Christian and Non-Christian parents did not even choose denominational schools that they found attractive. The access criterion ‘school type’ thus clearly limits the choice options and access chances of some parents, leading to a reinforcement of religion-based school segregation.

In addition to these decisive criteria, other criteria, such as cases of hardship, siblings’ school attendance, kindergarten proximity, and the ‘balanced’ ratio of children’s first languages, must be considered by school principals as well. Both the unspecific definition of those criteria (‘cases of hardship’) and the rather vague instructions on how and in which order to implement them (‘must be considered’) not only provide schools with a certain leeway in weighing up priorities^7^ when it comes to the admission process. They also force school principals to fill these ambiguities with content and to find their own way of dealing with applications and rejections. For example, the Aspen School principal pointed to their decisional scope when weighing up different criteria (cases of hardship; siblings’ school attendance): *“Where you might say: the family has had two children with us. Although they are no longer here, we have a high level of trust in the family. There are special cases with room for negotiation*.” Considering previous research that shows that groups with fewer resources are significantly more dependent on organizations (Allard and Small, 2013), this can be a problem most likely affecting how school principals use their leeway. As revealed by Lipsky (1980), street-level bureaucrats tend to support clients who are likely to be successful and may favor those with similar ethnic backgrounds (p. 107–108), leading to the assumption that the fewer clear regulations, the more leeway front-line workers have and the more susceptible the system is to discrimination and disadvantage. The combination of fuzzy admission criteria and individual decisions and strategies may thus contribute to school segregation.

Moreover, our analysis shows that not only legal regulations (such as the (non)definition of admission criteria) influence school segregation, but also their concrete interpretation and application. The five Treefield principals interpreted the selection criteria in very different ways. There was disagreement – between the school principals as well as between schools and the schools department – about whether, in the case of over-enrollment, the distance to the nearest elementary school or to the nearest one of the selected school type (i.e., Protestant/ Catholic/non-denominational) was decisive. Thus, the different ways in which this criterion was interpreted by the principals clearly showed that even the legal criteria provide selectivity leeway that each school can use in the sense of producing its own (above-described) idea of a ‘good’ mix. Further disagreement related to the application of the criteria. While some schools used Google Maps to estimate the distance between a child’s home and the school, others used distance tables provided by the schools department. As past enrollment decisions concerned differences of just a few meters, the form of measurement can be quite decisive. Parents criticized the lack of transparency regarding the criteria applied. For example, one mother with a university degree who considered herself to be ‘middle to upper class’ complained about the vagueness and got in touch with the schools department for further information: *“No one was able to tell me what the closest school to us would be […] It was really weird.”* (Ina).

How strongly institutional players react and adapt to parents’ strategies (and vice versa) becomes also apparent in the following: Several school principals indicated that some parents – particularly those perceived to be socioeconomically privileged – had exerted pressure regarding the distance between home and school. As a direct reaction to this, one school further checked whether the second choice indicated by parents referred to a school with available places in order to reduce over-enrollment and avoid conflicts with parents perceived to be articulate and assertive. These parents were then the first to be turned down (and consequently disadvantaged in their first choice). At the same time, several parents were well aware of the strategic importance of their first and second choices. Consequently, some socioeconomically privileged, non-denominational families deliberately indicated as their second choice not the school they liked second best, but a denominational school far from their home, in the expectation that this would increase their chances of their first choice being accepted: *“We specified school xy as our second choice. It’s a Catholic primary school quite far away. Because of the distance, we were pretty sure that we would fall off the grid. We hoped that this would bring us closer to our first choice.”* (Ina). This strategic thinking was particularly evident among middle-class parents as the one quoted or Yasemin, a mother able to navigate the German education system and using various information channels (open days, websites, conversations with other parents and school principals) to get a good picture of available options.

The above-mentioned example of giving in to parents’ pressure already indicates that the vagueness of admission criteria and the subsequent room for maneuver is not necessarily in the interest of school principals. Rather, it leads to frequent complaints (and even lawsuits) by parents about rejected enrollments and, subsequently, ends up in a considerable amount of extra work for the principals and a high level of uncertainty about the legally complex admission process. Considering this complexity, the principals did not feel sufficiently supported by the schools department since they were ultimately the ones responsible for redistributing children. One described the coordination meetings attended by all principals in the neighborhood as follows:

*“[…] you have to imagine it a bit like being in an Arab bazaar. Everyone shouts out what he or she has to offer: ‘I’ve got five to give away, I could take in four’. ‘Where do they live? Oh there. Are they Protestant? […] No. Then it won’t work there’. And then the kids get haggled over, quite terribly. You can’t listen to that as a mother. It’s quite scary.”* (SP Maple School)

These ambiguities and irregularities in dealing with enrollment illustrate that the process is perceived as a form of bargaining. The implicit leeway provides room for unequal treatment and discriminatory practices (Lipsky, 1980; Allard and Small, 2013) and can subsequently reinforce school segregation. Some parents seem to be aware of this. Of the 32 families who participated in the survey and were not accepted by their first-choice school, six indicated that they suspected this was for religious reasons, two for cultural reasons, and one for its social status. Dila, a Turkish parent with a higher education background, criticized the lack of ethnic heterogeneity in her child’s school and frankly suspected schools of playing an active role in selection: *“I do feel that the elementary schools have the luxury of being very selective about who they want and who they do not want.”* (Dila) Others, like Sinem, a lawyer with a Turkish background and own experiences of discrimination as a schoolchild, experienced the one-to-one meetings with school principals, for example on open days or enrollment day, as a kind of test of their ‘social fit’ and abilities:

*“It’s a bit like a court case. There sits the judge who is also only human. And he tries to get an idea of all participants in the proceedings. The more eloquent they are and the more expertise they can present, the more they impress him. And that’s exactly how it is, I think, in schools […] I think every school has [its] policy, enforcing consciously or unconsciously.”* (Sinem)

Well aware of her own agency in the admission process, Sinem knew how ‘to impress’ the principals in those meetings in order to get her child into their preferred school. Other (high-qualified) parents acted similarly. Amira, a mother with (third-generation) Bosnian roots and a university degree, and guided by own experiences of discrimination at school and university, described her influence on her child’s enrollment in the school’s after-school program - which for her was crucial in choosing a school:

*“Yes, and then it went back and forth for I do not know how long. I set all levers in motion, called around like crazy. With the principal here, the principal there … And then somehow two weeks before the start of school they said: Yes, he has a place in the afternoon care.”* (Amira)

Although the interviews provided no direct evidence of parents’ individual knowledge and negotiating skills helping determine enrollment decisions, the described leeway in applying the criteria shows that this is taken into account by parents in their school choices. Accordingly, parents rated their respective chances (next to official criteria such as their place of residence) also in relation to their ‘eloquence’, educational background or, for example, their involvement in religious communities, already anticipating their chances at the respective schools.

Interestingly, statements by the schools department showed that this leeway for school principals is not seen as any great problem. Rather, it is explicitly desired. “*Principals can assess the different schools and know a child might be a good fit for this or that elementary school*” (schools department representative). The quote illustrates that the principals’ discretion is explicitly welcomed, primarily since it explicitly enables those who are apparently expected to have the most in-depth knowledge about schools and the respective children – i.e., school principals – to make the final decision. Furthermore, it enables principals to decide not solely on the basis of ‘hard facts’ but also of a child’s and family’s social fit – and thereby, to ‘keep the business running’ as illustrated by previous studies on the discrepancy between written policies and their concrete implementation by frontline workers (Karakayalı and Zur Nieden, 2019).

When not all children are allocated to a school in the aforementioned coordination meetings, the schools department steps in, giving advice to parents and responding to complaints. Moreover, reacting to increased pressure from affluent parents in recent years, the schools department repeatedly decided to increase the enrollment capacities of popular schools to be able to offer more parents a place there instead of being forced to send them to the less popular schools. In the 2021/22 school year, an additional first class was created in three schools in the neighborhood – all with a comparatively privileged composition. For two of them, this even meant that they exceeded the city’s maximum enrollment capacity for elementary schools. Increasing enrollment capacity in popular schools is thus a clear strategy chosen by schools departments to avoid conflicts with those parents who, in order to ensure their child gains access to the ‘best’ school, might take legal action against the administrative decision.

Although less explicit than in the Malmö study (Voyer, 2019), the case of increased enrollment capacities showed how administrative decisions can implicitly perpetuate school segregation. Though it might seem counter-intuitive that such measures do not lead to a more mixed school composition, the quantitative online survey showed that one of the newly created classes was almost exclusively composed of children from high-income families from a new housing development, allowing them (as revealed in the interviews) to avoid a nearby school with a ‘negative’ social and ethnic composition. It can therefore be assumed that, here too, a form of selective admission by the schools took place. Thus, increasing the number of students at popular schools does not necessarily seem to increase the access chances of children with a migration background or children from low-income families. The fact that, at least in the past, some schools must set up additional classes, while capacities at others are under-utilized, can be criticized as an inefficient use of existing resources. Even more importantly, however, capacity planning based solely on the will of parents endowed with high cultural capital (such as German language proficiency or knowing how to navigate bureaucracy) does nothing to counteract existing inequalities regarding access to schooling.

### 4.3. Profiling and information policies

School principals can also shape their respective school compositions outside the enrollment process (Jennings, 2010; Ramos Lobato, 2017; Voyer, 2019). The city’s schools inspectorate mentioned, for example, the way individual schools developed their profiles or implemented their external communication and information policies. These involved, for example, (extra-)curricular activities, decisions on (increasing) the capacity of a school’s afternoon care programs^8^ or cooperation with (sports) clubs in the neighborhood. All these actions are taken in fulfilment of a school’s aim to attract the desired parental clientele (Van Zanten, 2005) by anticipating (middle-class) parents’ preferences and needs. They thus have a decisive influence on parental school choice and indirectly contribute to unequal access to educational resources (Kosunen et al., 2020).

The profiles of the five schools are only briefly described on the respective websites and are not clearly distinguishable at first glance. All five offer extracurricular activities and initiatives offered by external partners (e.g., a choir project). The majority also cooperate with local sports clubs, allowing them to use their sport facilities and take up their offerings. However, parents also perceived differences in the profiles. Josephine, one Chestnut School parent with a high socio-economic status and a teacher herself, described the profile of the more popular Maple School as follows:

*“They have, for example, a fencing club and a golf club […] These are expensive hobbies and therefore simply do not appeal to everyone or perhaps exclude some people. We are more into soccer which everyone can do somehow.”* (Josephine)

The quote makes clear that parents are not only aware of minor differences in school profiles, e.g., extracurricular activities, but that these can also be decisive for their school choice. At the same time, it can be assumed that these offers deliberately target a specific clientele, i.e., that (middle-class) parental preferences are anticipated by the schools when offering specific services and profiles. Interestingly, parents do not seem to assess these offers and activities exclusively under pedagogical and/or educational criteria. Rather, certain activities (such as golf or fencing) seem to be assigned to certain social groups, meaning that parents seem to understand very well whom the offers target. Consequently, school choice is also dependent on parents’ ability to fit in socially. Both the interviews and the online survey illustrate that parents with lower incomes are to some extent even deterred by profiles oriented, albeit implicitly, towards higher-income families or particularly attractive to middle-class families. Their fears and concerns relate to the high financial costs potentially involved for certain school activities and the associated social pressure and the implicit understanding of middle class as a norm (see also Ramos Lobato et al., 2018; Ramos Lobato and Groos, 2019).

The effect of school profiles on parents’ school choices works both ways and thus leads to self-reinforcing effects, partly for purely financial reasons. Despite the basic funding that all public elementary schools receive, they have different financial resources at their disposal, depending on their socio-economic composition as well as the level of support from the respective parents’ association. For example, the principal of Maple School, where high-income parents are overrepresented, describes the advantages of afternoon care programs sponsored by its strong parents’ association: “We purchased displays for six classrooms last year for 18,000 euros. That was no problem for us at all.” A hockey class and recording studio also figured among the special features of the resource-rich school. By contrast, the more under-resourced Poplar School relied heavily on external funding. The effect of school activities on parents’ school choice (and vice versa) thus reinforces educational inequalities and school segregation. Nevertheless, there are no cross-school efforts to align activities with a view to reducing or counteracting school segregation.

Another example of the influence of school profiles on student composition can be seen in the design of the afternoon care program. Differences related not only to the number of places offered, but also to more qualitative dimensions such as (more or less restrictive) regulations on pick-up times. While Poplar School can usually offer free afternoon care places, the popular Chestnut School has been unable to meet demand in recent years – a fact openly communicated by the school with the underlying intention of reducing the extra work caused by afternoon care over-enrollment. Particularly for full-time working households or single parents who cannot afford childcare outside school, afternoon care capacities can play a key role in school choice. These less privileged groups were therefore deterred from opting for Chestnut School, instead going for Poplar School.

Parental choice is not only influenced by school profiles, but also by how these profiles are marketed. *“We strive for a good tone, yes, we are like a service company. We provide information, we react very quickly, we try to offer, we try to find solutions.”* (SP Aspen School). This is a quote from the Aspen School principal who suspects that their (deliberate) communication strategy has led to increasing numbers of highly-educated families. At the same time, however, communication strategies (consciously or unconsciously) can discourage certain groups of parents. One example of such a (consciously) selective information policy targeting certain social groups concerns the messaging of the two denominational schools in our case study area. While the popular denominational Chestnut School seems to downplay its Catholic profile when addressing non-religious but socio-economically more affluent households, the opposite seems to be the case with less ‘desirable’ parents. A low-income Muslim family, for instance, recalls a conversation with the principal, in which the principal emphasized the mandatory Catholic offerings and thereby tacitly suggested that this was not the right school for their child. This illustrates how a different profile (in this case, a denominational profile) can be communicated in different ways, depending on the addressees and the schools’ ‘target group’ and thus illustrates that access to schools can (besides direct forms of discrimination such as access criteria) also be controlled and steered by subtle institutional practices.

The deliberate influence of a school’s communication strategy on parental choice points to the competition between the schools, as reported by Alexandra:

*“My friend told me […] that she […] was unsure whether to enroll her child in Poplar School or Chestnut School. The Chestnut School principal told her […] she needed to know whether she wanted a [full-time] afternoon care place [at Poplar School] or a ‘good’ school [Chestnut School] for her child.”* (Alexandra)

According to Alexandra, who herself has worked in the education sector for ten years in a deprived area of the city, the school principal not only suggested that there were qualitative differences between the schools in the neighborhood, but also deliberately played on the concern of many parents to choose the ‘best’ school for their child (Ramos Lobato, 2019; Ramos Lobato and Groos, 2019; Breidenstein et al., 2020). This more or less open form of ‘poaching’ students once again illustrates the non-existence of overarching guidelines on school segregation and subsequent cooperative models for distributing students (equally) among the schools in question.

The lack of inter-school strategies to avoid school segregation is also evident in the communication channels chosen by schools. A very important information platform for parental elementary school choice are kindergartens, which often cooperate with certain elementary schools and thus enable schools to present themselves in the context of ‘taster days’. Kindergartens are settings where parents exchange information about the transition to elementary schools and where educators are frequently asked by parents for their assessment of the choice of the ‘best’ school. As shown in previous studies (Ramos Lobato, 2019), targeted cooperation with selected kindergartens influences the composition of the respective elementary school, meaning that segregation already starts at preschool level (Groos et al., 2018). As kindergarten composition and school composition are linked, it seems to be quite relevant for schools to cooperate with a certain type of kindergarten. However, similar to the above-mentioned dimensions, the expert interviews (two of which were conducted with kindergarten heads) reveal that the cooperation between schools and kindergartens is also not a subject of inter-school discussions – despite its great potential for mitigating school segregation. Rather, cooperation is established through personal contacts between heads of schools and kindergartens.

Overall, this scrutiny of school profiles and information policies revealed that many opportunities to mitigate school segregation remained untapped.

## 5. Conclusion

In line with a series of studies on the influence of parental choice on school segregation, our study illustrates how institutional norms and routine practices in the educational sector help shape access to schools and, subsequently, contribute to school segregation. While most studies on institutional discrimination focus on the UK, the US, and Australia (Lewicki, 2022), our case study sheds light on organizational routines in Germany – an interesting educational context where accountability levels and the frequency of mandatory standardized tests at schools are far less pronounced than the OECD average (OECD, 2016).

A key finding of our research is the close interplay between the practices of institutional players (the schools department and school principals) on the one hand, and parental choice on the other, in exacerbating school segregation. Until now, this interplay has been under-researched in international studies. We thus call for a stronger contextualization of street-level bureaucrats’ practices in order to analyze their logics of action not only against the background of their own organizations, but also beyond (Lipsky, 1980). We identified three dimensions of institutional guidelines and practices decisively shaping school segregation that can partly be considered as (in)direct forms of institutional discrimination: (1) the lack of school guidelines for dealing with segregation; (2) derived from this, the enrollment process; and (3) a school’s profiling and information policy targeting specific groups of parents.

First, there are no guiding principles on the social composition of schools. Rather, school segregation is unilaterally interpreted by the school principals and schools department representatives as a challenge to be overcome by those schools in which children perceived as ‘problematic’ are overrepresented. The fuzzy institutional guidelines give local schools a certain leeway for achieving a ‘good’ mix and differentiating between ‘good’ and ‘bad’ pupils. The institutionally embedded organizational practices (Allard and Small, 2013) and the reproduction of whiteness as a norm, as described by Ahmed (2012), are often implicit and subtle, yet a crucial part of institutional discrimination.

Second, lacking or fuzzy guidelines are also a characteristic of the admissions process. The insufficient clarity of enrollment criteria gives principals a certain leeway in interpreting and prioritizing different criteria. In this respect, both local school principals and the schools department have an implicit understanding of what a ‘good’ mix looks like. While a strong presence of children from white, ‘assimilated’ or socioeconomically privileged families is seen as an indicator of a ‘good’ mix (Jennings, 2010; Voyer, 2019), racialized children, children with a foreign native language, and children from socioeconomically disadvantaged families are problematized and associated with more intensive care, and thus a greater workload (Van Eycken et al., 2022; Dursun et al., 2023). The power of more privileged and demanding parents and their impact on institutional players’ practices is also illustrated by the fact that schools try to anticipate and/or avoid potential complaints from this group, for example by establishing more classes at more popular schools (for them).

Third, school profiling and information policies reinforce school segregation: on the one hand because certain school profiles and offers are very explicitly oriented towards the needs of specific parents; on the other hand because certain parents are discouraged by them, e.g., socioeconomically disadvantaged parents by costly extracurricular activities. At the same time, parents try to anticipate their chances of being accepted, weighing up school profiles and accordingly not enrolling their children in schools where they expect access to be more difficult (e.g., a school with a denominational profile).

One key finding of our study is that school segregation affects all schools in a neighborhood – regardless of their composition and popularity. The partly implicit, partly explicit pursuit of a ‘good’ mix at schools structurally disadvantages certain families and thus must be understood and politically discussed as indirect discrimination. The goal should be for all schools (including public denominational schools) to reflect the social composition of their surrounding neighborhood and grant equal access. As other international studies have already shown, this requires (at least in the German case) more distinct discrimination-sensitive guidelines, more inter-school cooperation, as well as greater enrollment transparency (Baysu et al., 2022; Boterman and Ramos Lobato, 2022a; Bonal and González Motos, 2023). Regarding the latter, an education reform in Amsterdam serves as an interesting example. In 2014, the school boards decided to implement a centralized enrollment system not only modifying parents’ potential choices by coupling them to their place of residence, but also making the admissions process more transparent and predictable. By reducing direct competition between schools, limiting their scope of discretion in admitting pupils, and reducing the negotiating space for parents, the reform may already have partly defused segregation (Boterman and Ramos Lobato, 2022a,b). The urgency of such institutional change is evident, not least in the context of the current influx of Ukrainian refugees into many European cities. Without shared guidelines and cooperation, there is a risk that school segregation will become even more pronounced.

Based on our study, we see a great need for further research into the interactions between the individual and institutional levels in the school admissions process. With parents with social characteristics such as low educational attainment and a non-German background under-represented in our study, a more targeted focus on these and other characteristics from an intersectional perspective would help further uncover the influence of institutional players and institutional discrimination on more vulnerable groups of parents and how groups are racialized as ‘other’ (Lewicki, 2022).

Furthermore, a comparative perspective on the role of school principals and their agenda-setting in different education systems (with varying degrees of school autonomy and accountability) would help better understand the role of accountability in guiding their strategies as well as inter-school competition. However, the local educational institutions and policies considered in our paper are just one aspect of addressing school segregation. Segregation between schools is also the result of a complex interplay between different levels of governance (Boterman and Ramos Lobato, 2022a), with local-level access opportunities and transparent procedures framed by educational policies at regional and national levels. In the future, therefore, further analysis is needed to take greater account of these institutional levels, in addition to parental choice decisions and the pre-structuring of school composition through residential segregation. Furthermore, future studies could make greater use of the perspective of institutional discrimination for international comparative studies. In this way, the role played by different national education or integration policy regimes and organizational settings in reinforcing inequality can be explored.

## Data availability statement

The raw data supporting the conclusions of this article will be made available by the authors, without undue reservation.

## Ethics statement

The funding institution, the German Research Foundation, approved the funding of our project and classified a separate ethical approval as not necessary. The study was conducted in accordance with the local legislation and institutional requirements. A thorough assessment of the research consequences and the evaluation of the respective ethical aspects was carried out. Hereby, we followed the guidelines for good scientific practice of the German Research Foundation. The participants provided their written informed consent to participate in this study and for the publication of anonymized data included in this article.

## Author contributions

IRL, AG, and HH contributed to the conception and design of the research study, participated all in each step of the research process, and participated in the interview analysis. The expert interviews were conducted by IRL, AG, and HH. The interviews with the parents solely by AG. AG wrote the first draft of the manuscript, while IRL and HH did significant revisions, particularly regarding the theoretical section. All authors contributed to the article and approved the submitted version.

## Footnotes

^1^In consultation with the education department, we decided against publishing the name of the case study city. We instead use a pseudonym for the district and schools.

^2^In Germany, municipal statistics derive a person’s ‘migration background’ from the combination of the characteristics ‘second nationality’, ‘origin of immigration’, ‘type of German citizenship’, and ‘country of birth’ (Städtestatistik, 2022). According to the official statistics in Germany, a person has a migration background if he/she or at least one parent was not born with German citizenship. The container concept of ‘migration background’ has only limited explanatory power in discrimination research, as the risk of being discriminated against is distributed unevenly within this group (e.g., according to skin color or religion) (Scherr et al., 2017, p. 465). Thus, we are aware of the justified analytical and normative shortcomings of working with this category (*cf.* Fachkommission Integrationsfähigkeit, 2020, p. 218f). Nevertheless, we use it as an approximation of ethnic segregation, as no data on other categories of analysis relevant to discrimination and racism is available.

^3^Since neighborhood-level data on the receipt of social welfare is not available, we use the exemption from paying for learning materials as a proxy for the proportion of students from low-income families. According to the city’s education department, households that receive social welfare, for example, can be exempted from paying for learning materials.

^4^A detailed list of all parents interviewed for this study can be found in the Appendix as supplementary material (Supplementary Table 2).

^5^All names used in this article are pseudonyms.

^6^The introduction of a school-specific social index (Schräpler and Jeworutzki, 2021), which could lead to an increase in positions for social workers, was only announced after the interview, in April 2021 for the 2021/2022 school year.

^7^The regulation states that “[The] school principal shall take into account cases of hardship and shall use one or more of the following criteria (…) for the admission decision: 1. siblings, 2. routes to school, 3. attendance of a preschool near the school, 4. balanced ratio of girls and boys, 5. balanced ratio of pupils of different native language.”

^8^Traditionally, German elementary schools provide schooling solely until lunchtime. In recent decades, there has been an increase of afternoon care programs, though in many cities there are just not enough places for all elementary school children.
