# Supplementary material for: “The kids get haggled over”: how institutional practices contribute to segregation in elementary schools
Source: Front Sociol. 2023 Oct 31;8:1250158. doi: 10.3389/fsoc.2023.1250158 (PMC10644677; doi:10.3389/fsoc.2023.1250158)
Supplement: Supplementary file 1 [file Table_1.docx]

# Supplementary material

# Table 2: List of interviewed parents

|  | **Pseudonym** | **Gender** | **Educational attainment** | **Ethnical/racial self-identification** | **Religious beliefs** |
| --- | --- | --- | --- | --- | --- |
| 1 | Nina | female | n.a. | n.a. | n.a. |
| 2 | Meral | female | University of applied sciences entrance qualification | German-Iranian | Non-denominational |
| 3 | Josephine | female | PhD student | German | Non-denominational |
| 4 | Daniela | female | University degree | German | Roman-catholic |
| 5 | Katharina | female | No high-school diploma, apprenticeship | German | Protestant |
| 6 | Kimberly | female | PhD | White, mother US-American | Christian |
| 7 | Nadine | female | University of applied sciences entrance qualification | White, grandparents from Yugoslavia | Protestant |
| 8 | Lena | female | PhD | White German | Atheist |
| 9 | Ina | female | Masters's degree | White | Atheist |
| 10 | Jessica | female | PhD | German | Protestant |
| 11 | Daniel | male | University degree | White European | Non-denominational |
| 12 | Luise | female | Master's degree | German | Catholic |
| 13 | Charlotte | female | Master's degree | White German | Catholic |
| 14 | Dila | female | University education | German-Turkish Swabian | Muslim |
| 15 | Simone | female | University degree | European | Christian |
| 16 | Sinem | female | PhD | German-Turkish | Alevist |
| 17 | Stefanie | male | Master's degree | White Central-European | Non-denominational |
| 18 | Karl | male | University degree | German | Non-denominational |
| 19 | Ellen | female | University degree | German | Protestant |
| 20 | Sabine | female | University degree | n.a. | Protestant |
| 21 | Isabelle | male | No high-school diploma | n.a. | Protestant |
| 22 | Ines | female | High-school diploma | n.a. | Non-denominational |
| 23 | Marie | female | University degree | n.a. | Protestant |
| 24 | Julia | female | University degree | German | No |
| 25 | Melek | female | University degree | Turkish-German | Alevist |
| 26 | Nicole | female | University of applied sciences degree | White German | No |
| 27 | Sonja | female | University degree | n.a. | No |
| 28 | Akosua | female | No high-school diploma | Ghanaian | Christian |
| 29 | Tabea | female | University degree | German | Catholic |
| 30 | Miriam | female | University degree | White German | Protestant |
| 31 | Franziska | female | University degree | German | Non-denominational |
| 32 | Sandra | female | University of applied sciences entrance qualification | Human | Non-denominational |
| 33 | Annabell | female | University of applied sciences degree | White German | Protestant |
| 34 | Felix | male | University degree | n.a. | Protestant |
| 35 | Hilal | female | High-school diploma, apprenticeship | Turkish | Muslim |
| 36 | Natalie | female | n.a. | n.a. | n.a. |
| 37 | Amira | female | University degree | Migration background | Muslim |
| 39 | Sebastian | male | University degree | German | Protestant |
| 40 | Defne | female | University degree | Multicultural | Non-denominational |
| 41 | Robert | male | University degree | German | Protestant |
| 42 | Caroline | female | PhD / university degree | German | Catholic |
| 43 | Tanja | female | University degree | n.a. | Non-religious |
| 44 | David | male | University degree / university degree | n.a. | Protestant |
| 45 | Alexandra | female | University degree | n.a. | Non-denominational |
| 46 | Annika | female | University degree | German (migration background "on paper") | Non-denominational |
| 47 | Katja | female | University degree | German | Catholic |
| 48 |  |  | University degree | n.a. | Catholic |
| 49 | Sabrina | female | University of applied sciences degree | White German (migration background in the family) | Catholic |
| 50 | Bianca | female | University degree / university degree | n.a. | Protestant |
| 51 | Martina | female | University of applied sciences degree | n.a. | Protestant |
| 52 | Cornelia | female | Apprenticeship | n.a. | Catholic |
| 53 | Yasemin | female | University degree | Migration background | Catholic |
| 54 | Adam | male | No high-school diploma | n.a. | Muslim |
| 55 | Kübra | female | University of applied sciences entrance qualification | n.a. | Muslim |
| 56 | Jennifer | female | High-school diploma, apprenticeship | German-Italian | Catholic |
